# My Experience in Stammering and Its Cure

**Published:** 1882-01-20

**Authors:** Thomas S. Gallaher


### MY EXPERIENCE IN STAMMERING, AND ITS CURE.

BY THOS. S. GALLAHER, M. D.

From my earliest recollection I stammered. My parents insisted that I had acquired it from a neighboring lad who was my constant companion in early years; but of this I cannot speak. It might have been as they said. All I know is that I was a great stammerer for years and years, and nothing appeared to do me any good. The more I tried to speak the greater was my impediment in speaking. At the age of seven, my father died and I was placed in a country village with an uncle who was a physician. My uncle was a very severe man and in his severity tried all means to cure me of the unfortunate habit. I was coaxed and bought, treated at times with kindness, and at others with severity, all to no purpose; finally he resorted to the rod. Of this weapon I frequently felt its stinging strokes till my back was sore and in stripes from its use. All the whipping and ill treatment I received on this account had no more effect in curing my habit than if I had been let alone. Indeed it served to increase the difficulty. Fear of stammering in the presence of my superiors appeared to lessen the co-ordinating influence over the muscles of articulate speech; hence the difficulty was increased rather than relieved. After living in the country nearly six years, I came to Pittsburg and remained till the great fire of 1845, with my maternal grand-parent. I was then 14 years old. I brought my stammering propensity with me. In fact I was worse than when I went to reside with my uncle. My grand-parent was a kind and forgiving old gentleman who seldom found fault with anything, especially with me for my misfortune. Under his gentle care I improved somewhat in my method of speech. I did not fear him and therefore could speak in his presence without so much embarrassment. This was really felt as a relief. However, I still stammered badly, especially if made angry or was in haste to communicate my ideas. Often have I wept and sorrowed at not being able to converse with my fellow creatures in the plain, simple style I saw others do, All professional callings appeared to be closed against me. All avenues for success in business circles did not seem to be open to me. An occupation of manual labor, which did not require the use of language, seemed alone suited to my condition. I would say here that I could sing with as free a voice as anybody, and could swear with as much freedom from impediment as any one could wish. But when I decided to communicate my thoughts in common language my efforts failed and I was often overwhelmed with shame.

This was my condition and such were my thoughts and feelings till I was 18 years of age. At this period I was induced by a friend to join what was called at the time, a debating society. It was composed of a party of young men mostly, if not wholly, mechanics, who had united themselves in a body for mutual improvement. They met weekly, had declamations, read original pieces, and debated the common questions of the day. They met during the winter alone.

My first effort in this society at debating was a total and complete failure. I stammered and stuttered so badly that I brought upon myself, I feared, everlasting disgrace. A few words of encouragement however, soon healed my wounded feelings and determined me to try to regain my lost laurels. The next appointment was for declamations. In this the success was but little better than the first. And so I continued from week to week, as my time came around, to fulfil the appointments. In a short time I found that I could speak with less difficulty when perfectly free from all fear and confusion. The least occasion that caused confusion would at once start the impediment. After a time, on learning my piece perfectly by note, standing in an erect position, perfectly quiet, making no gestures, fixing my eyes steadily upon some point above the chairman, and speaking in a loud voice as though the speech was addressed alone to forest trees, and not to men, and perfectly unconscious of any one being present, I was able to declaim with almost perfect freedom of speech. In thus declaiming, should the eyes be taken off the point at which they were directed, and glanced upon the president or any of the members, or should any motion of the body supervene or a single gesture be made, or any interruption of the performance occur, my self-reliance would leave me, confusion take its place, and a return of the unhappy stammering would certainly follow. This gained confidence in myself, threw aside all fear of speaking in public, and after many trials, in which there were many failures as well as triumphs, finally overcame all difficulties and could speak with considerable fluency and ease. In fact a cure of the stammering was effected. It required about two years to complete the cure. To this day I remain free from the trouble.

The stammerer is required to hold his breath so much while speaking, and to respire with such regularity that the heart in time becomes affected with hypertrophy and dilatation due to obstructed circulation from irregular and spasmodic breathing. I suffered somewhat from this cause.

From my individual experience I am satisfied that the seat of disorder is in the nervous centers controlling the muscles of articulate speech, as well as those of respiration. These centers appear to be influenced by the various mental operations already suggested by which the proper transformation of thought into language is interfered with. The disease is closely allied to chorea.

I have now given my experience in stammering, and hope to have made plain the method of its cure. I have given it in the interest of a very large class of people who are subject to this infirmity, a class whose hopes and prospects have been sadly curtailed by the infringement upon the right of free and untrammeled speech.

To conclude, I would repeat that the disease has its origin in fear and confusion of thought, and must be remedied by such exercises as will produce free, independent and uncontrolled speech.—[Pittsburg Medical Journal.

